# Near full-length HIV-1 subtype B sequences from the early South African epidemic, detecting a BD unique recombinant form (URF) from a sample in 1985

**DOI:** 10.1038/s41598-019-42417-1

**Published:** 2019-04-17

**Authors:** Adetayo Emmanuel Obasa, Susan Engelbrecht, Graeme Brendon Jacobs

**Affiliations:** 1 Division of Medical Virology, Department of Pathology, Faculty of Medicine and Health Sciences, Stellenbosch University, PO Box 241, Cape Town, 8000 South Africa; 2 National Health Laboratory Services (NHLS), Western Cape Region, Tygerberg Hospital, Cape Town, South Africa

**Keywords:** Whole genome amplification, Epidemiology

## Abstract

HIV-1 subtype C is the most prevalent subtype in South Africa. Although subtype B was previously detected in South Africa, there is limited sequence information available. We characterized near full-length HIV-1 subtype B sequences from samples collected at the start of the South African HIV-1 epidemic, in the 1980s. Five samples were analysed by PCR amplification, Sanger DNA sequencing and phylogenetic analyses. The viral genomes were amplified in two overlapping fragments of 5.5 kb and 3.7 kb. The sequences were subtyped using REGA version 3.0, RIP version 3.0 and jpHMM. Maximum Likelihood phylogenetic trees were inferred with MEGA version 6. Four HIV-1 patient sequences were subtyped as pure HIV-1 subtype B. One sequence was characterized as a novel HIV-1 subtype B and D recombinant. The sequences clustered phylogenetically with other HIV-1 subtype B sequences from South Africa, Europe and the USA. We report the presence of an HIV-1 subtype B and D recombinant strain detected in the beginning of the epidemic. This indicates that viral recombination events were already happening in 1985, but could have been missed as sequence analyses were often limited to small genomic regions of HIV-1.

## Introduction

Since the beginning of the HIV-1 pandemic more than 35.4 million (25.0–49.9 million) people have died of AIDS-related illnesses^1^. South Africa experiences the largest HIV-1 epidemic with 7.2 million infected individuals^2^. The first HIV-1 cases in South Africa were reported in 1983 and the infections were confined to men who have sex with men (MSM) and bi-sexual risk groups^3^. The viral sequences between 1984 and 1990 were characterized as HIV-1 subtypes B and D^4–6^. The spread of HIV-1 subtype C in the heterosexual population in South Africa has been on the increase since it was first identified in the late 1980s^7^.

There is a total of 182 short fragment HIV-1 subtype B sequences from South Africa available in the Los Alamos HIV database (http://www.hiv.lanl.gov), accessed in September 2018. The majority of these short fragment sequences belong to the envelope (*env*) coding region and the *gag*-*pol* region. Out of these, only five subtype B near full-length genome sequences from South Africa have been published: R84 (FJ647145)^8^, TV016 (KJ948656)^9^, TV047 (KJ948657)^9^, TV1057 (KJ948660)^9^, and 03ZAPS045MB2 (DQ396398)10^10^. Four of these sequences were characterized in our laboratory. Patient R84 was a MSM sampled in 1985^8^, TV016 was a heterosexual male sampled in 1988, TV047 was a heterosexual male sampled in 2000 and TV1057 was an MSM sampled in 2002^9^. Wilkinson *et al*.^9^ described these sequences, while Rousseau *et al*.^10^ described patient sequence 03ZAPS045MB2, a female sampled in 2003. Thus, there are limited information available for HIV-1 subtype B near full-length sequences from the early epidemic in South Africa. In Africa, there is only one published near full-length HIV-1 subtype B sequence OYI 397 from Gabon, described by Huet *et al*.^11^. Patient sequence OYI_397 was sampled in 1988 and the risk factor, gender and patient ethnicity were not recorded^11^.

The objective of this study was to characterize HIV-1 subtype B near full-length virus sequences from the early South African epidemic from 1985–1987. This study forms the basis for continued research in our attempt to reconstruct the epidemiology and evolutionary history of HIV-1 in South Africa.

## Results

### Patient demographics and virus isolation

The demographic and clinical data of the patients are summarized in Table 1.

**Table 1:** Demographics and clinical data of patients.

Patient	Age	Risk factor	Sample date	Clinical symptoms	Viral phenotype	RT activity (cpm/ml)
R68	24	MSM	1985	Symptomatic	SI	569940
R459	34	MSM	1987	Symptomatic	SI	1607500
R526	32	Bisexual	1987	Symptomatic	SI	36227
R605	29	Bisexual	1985*	Asymptomatic	Uncultured	Uncultured
R1296	48	Bisexual	1986	AIDS	NSI	951000

^*^Blood sample dated 1985, **MSM**: Men who have sex with men; **SI**: Syncytium Inducing; **NSI**: Non-syncytium Inducing; **RT**: Reverse Transcriptase.

Virus isolation was identified by cytopathic effect in the co-cultures for both patients R68 and R459. The cytopathic effect consisted of large multinucleated giant cells with clear balloon-like cytoplasm for R68 and smaller slowly developing syncytia for R459. The co-cultures for R526 and R1296 were non-cytopathic and virus isolation was confirmed by the Reverse Transcriptase (RT) assay. The RT counts ranged from 36227 to 1607500 counts per minute per millilitre (cpm/mL). We received a blood sample from R605 and no virus isolation was done.

### PCR amplification and near full-length genomic sequences

All PCR amplicons were positive for both fragments 1 and 2. Assembly of the overlapping sequence contigs resulted in the following sequences, relative to HXB2: R68 [832–9183 base pairs (bp)], R459 [849–9222 bp], R526 [814–9222 bp], R605 [850–9186 bp] and R1296 [1042–9219 bp]. The sequences resulted in the characterization of near full-length HIV-1 genomes spanning from the 5′ *gag* region through the 3′ U3 region. Open reading frames were identified for *gag, pol*, and *env* structural genes and for *vif, vpr, vpu, nef, tat*, and *rev* regulatory and accessory genes.

### Maximum Likelihood phylogenetic tree inference

**Figure 1:**
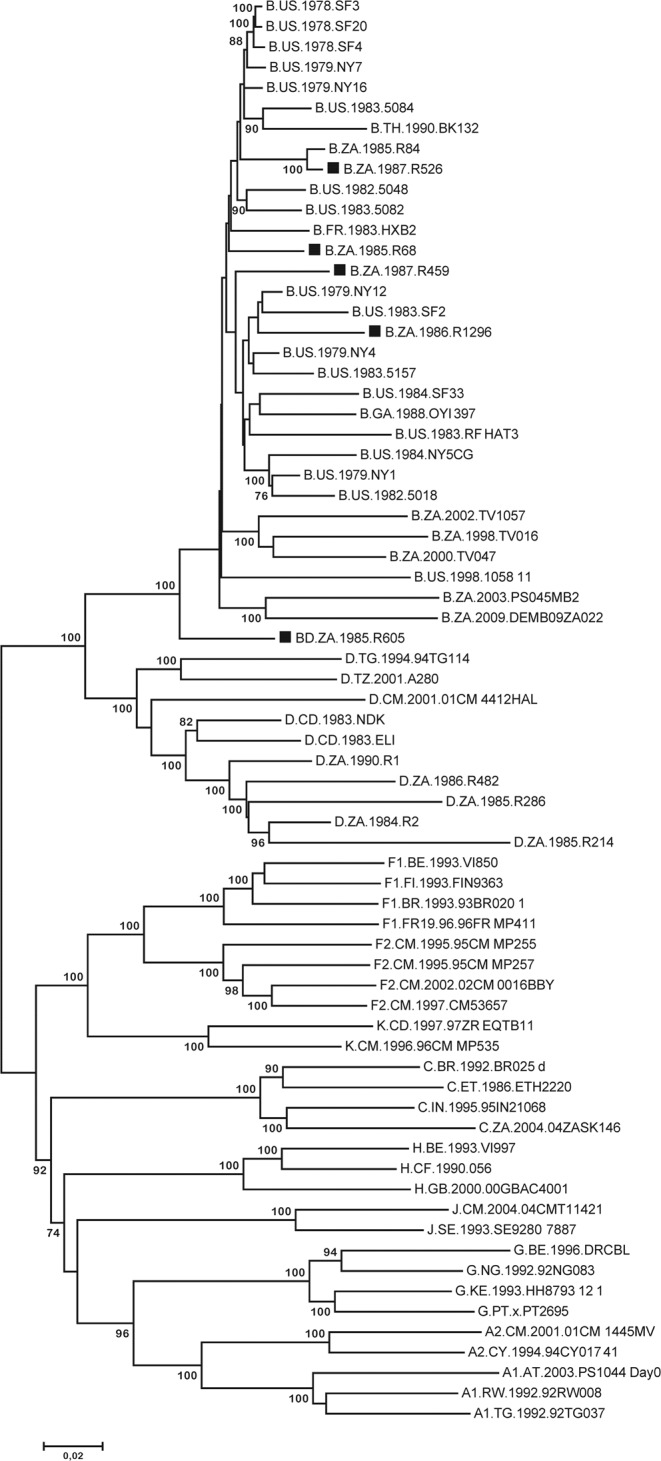
HIV-1 near full-length genome (NFLG) molecular phylogenetic analysis inferred by the maximum likelihood (ML) method. The ML phylogenetic tree inferred in MEGA version 6 contains the five new HIV-1 sequences (■), HIV-1 subtype B and D reference sequences, previously characterized sequences from South Africa and the HIV-1 Group M reference subtypes. The evolutionary distances were inferred using the general time reverse (GTR) model of nucleic acid substitution with an estimated Gamma shape parameter and invariant sites. The genetic distance is displayed in the scale bar at the bottom of the figure. Bootstrap values higher than 70% are indicated.

Maximum Likelihood (ML) tree topologies were inferred for the near full-length sequences [Fig. 1]. The best-fitting nucleotide substitution model was evaluated using MEGA version 6. Four out of five patient sequences clustered within subtype B reference sequences, while sequence R605 is seen as a HIV-1 subtype B outlier. Sequence R526 clustered with sequence R84 from South Africa with high bootstrap support. Sequence R68 and R459 clustered with strains from the USA. Sequence R1296 clustered with strains from San Francisco and New York.

### Recombinant analyses

**Figure 2:**
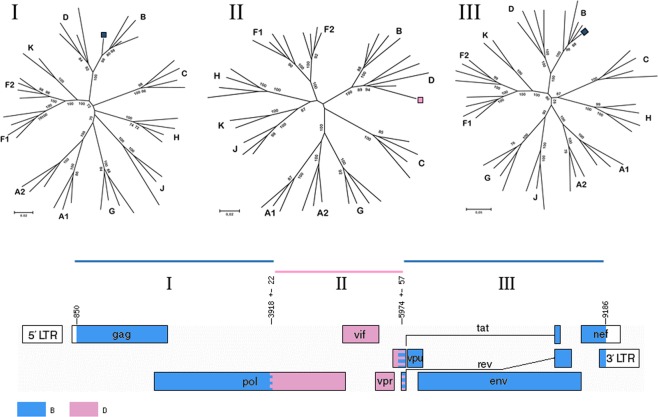
Subtyping analyses of the unique recombinant form (URF) of R605, a BD recombinant sequence according to jumping profile Hidden Markov Model (jpHMM), LANL and phylogenetic analyses. Phylogenetic analyses (ML tree) according to breakpoint located at position (862–3918 bp) in the *gag* - *pol* region clusters amongst subtype B sequences. ML tree according to breakpoint position (3918–5974 bp) in the *pol* – *vif* region clusters amongst subtype D sequences. ML tree according to breakpoint position (5974–9187 bp) in the *vpu* – *env* region clusters amongst subtype B sequences. In order to validate the sequences a total of 100 bootstrap replicates was carried to infer a ML phylogenetic tree.

A representation of the unique recombinant form between subtypes B and D of R605 is illustrated in Fig. 2. The following breakpoints were observed from the jpHMM analyses: *gag* – *pol* (862–3918 bp HXB2) subtype B, *pol* – *vif* (3918–5974 bp HXB2) subtype D and *vpu* – *env* (5974–9187 bp HXB2) subtype B. In order to validate the recombinant breakpoints, ML phylogenetic trees were inferred for each fragment.

## Discussion

In 1982 the first two HIV-1 patients were reported in South Africa in MSM patients. Both had contacts in the USA before developing AIDS^3^. In South Africa, two independent epidemics have been described^7^. The first was initially spread by MSM. This was later identified as HIV-1 subtype B and D^4–6^. The second epidemic started to expand in the heterosexual population as far back as early 1988, with seroprevalence data in blood donor populations from different race groups that showed a characteristic pattern. These independent epidemics are consistent respectively to global epidemiological patterns^12^. In addition, human migration is a major process that shaped the origin and dissemination of HIV-1^13^. Data generated by Wilkinson *et al*.^14^ indicate that in South Africa the bulk of viral introductions occurred during a period of socio-political change in the country (1980–2000)^14^. Based on our current knowledge South Africa experienced multiple introductions of HIV-1 from neighbouring countries towards the move to full independence in the 1990s, after the end of Rhodesian bush war^15^. The results generated from this study further strengthen the hypothesis of the presence of HIV-1 subtype B and HIV-1 subtype D amongst the MSM risk group at the beginning of the epidemic in the country. This may also explain the HIV-1 subtype BD URF detected and sequenced from the PBMC of patient R605.

In 2014, an unusual high HIV-1 subtype diversity was described in Cape Town^16^. This suggests that international tourism and MSM migration into the country, seeking constitution protection, might play a vital role to the MSM network in Cape Town^16^. Furthermore, there has been an increase in the number of subtype B cases spread through heterosexual and mother-to-child transmission. In this study, five new near full-length HIV-1 sequences were characterized: four subtype B’s (R68, R459, R526 and R1296) and one unique subtype B and D recombinant form (R605). The near full-length subtype B sequences included in this study were isolated from both MSM and heterosexual individuals. Patient R84 sampled in 1985 (GenBank FJ647145) is the only subtype B near full-length sequence which has been characterized from the early epidemic in South Africa^8^. This subtype B isolate, along with 5 subtype D viruses, were characterized in three separate studies, represents the only near full-length sequences of the early MSM HIV-1 epidemic within South Africa^4–6^. Three subtype B sequences have been described from the later epidemic: TV016 sampled in 1998 (GenBank KJ948656)^9^, TV047 sampled in 2000 (GenBank KJ948657)^9^ and TV1057 sampled in 2002 (GenBank KJ48660)^9^. In Gabon, OYI_397 sampled in 1988 represents the only other HIV-1 subtype B near full-length sequence published from Africa^11^. Although the infection occurred in the 1980s, analyses of early sequences are critical to understanding the time of origin of the early epidemic in the country^14^.

In the 1980s a minor subtype D epidemic was also present in South Africa^4,5^. According to the LANL HIV database (accessed 04 June 2018), there are 83 near full-length pure HIV-1 subtype D sequences described in the world and only 10 (12.04%) of these sequences were not from Africa. Loxton *et al*.^4^, and Jacobs *et al*.^5^, described five HIV-1 subtype D viruses from South Africa. Limited focus has been placed on this HIV-1 subtype D in this country. In 1997, HIV-1 subtype D was identified in a male MSM and one heterosexual patient through partial *gag* analysis^17^. Bredell *et al*.^18^ conducted a study where they identified a HIV-1 subtype C and D recombinant in a heterosexual through partial sequencing of *gag* and *env* gene. Furthermore, in the same study, they also identified HIV-1 subtype D in a male heterosexual through partial sequencing of the *gag* gene. There are currently 83 near full-length HIV-1 subtype D sequences in the Los Alamos Database, of which only ten were not from Africa. Before 1990 there was eight near-full-length subtype D sequences described. Four of these sequences, R2 (1984), R214 (1985), R286 (1985) and R482 (1986), were from South Africa^4^. The other four sequences were from the DRC: NDK (1983)^19^, ELI (1983)^20^, 84ZR085 (1984)^21^ and CDC Z2 (1985)^22^.

In this study, we identified an HIV-1 subtype BD Unique Recombinant Form (URF) from a patient sample obtained in 1985 during the start of the epidemic in South Africa. To the best of our knowledge, R605 is the first URF_BD described in the world. In South Africa, previous studies have identified the presence of URFs. In 2014, Jacobs and co-workers described the emergence of a possible URF of HIV-1 subtype B and C in South Africa^23^. In addition, the presence of recombinants AD (TV101) and AC (TV218) were also described. TV101 is the second AD recombinant described in 2015 and is closely related to AF457082 from Kenya^24^. The first AD recombinant characterized were from a South African patient that was infected via heterosexual contact in Kenya. Recombinant AC (TV218) was sampled from a 25-year-old female in Durban^9^. Two AC recombinant forms were also described by Rousseau *et al*.^10^ and Papathanasopoulos *et al*.^25^. The breakpoints (recombination events) in all four of the AC recombinant fragments differ and thus each one represents URFs. In addition, previous studies have described the presence of unique inter-subtype recombinants such as BC, BF, and AC in both the MSM and heterosexual populations^16,23,25^.

## Conclusion

With the advancement of molecular techniques and the availability of stored samples, we managed to characterize an HIV-1 recombinant sequence, sampled in 1985 in South Africa. Phylogenetic inference of five newly sequenced HIV-1 strains, identified subtypes B and the novel BD recombinant. Characterization of HIV-1 sequences during the early years were often based on short sub genomic regions. This most likely limited the identification of recombinant viral strains. Only near full-length genome analyses of HIV-1 can enable us to make accurate assumptions on viral recombination and pure subtypes. It is necessary to continue monitoring the evolution and spread of HIV-1 as understanding HIV-1 diversity can help us understand specific transmission patterns and help us understand how the virus continues to spread in South Africa and worldwide.

## Methods

### Ethics statement

This study was approved by the Health Research Ethics Committee of Stellenbosch University, South Africa (N15/08/071). The study was conducted according to the ethical guidelines and principles of the international Declaration of Helsinki 2013, South African Guidelines for Good Clinical Practice and the Medical Research Council (MRC) Ethical Guidelines for Research. A waiver of consent was awarded to conduct sequence analyses on these archived samples.

### Study design

HIV-1 strains from infected patients were isolated routinely at the Division of Medical Virology, Tygerberg Academic Hospital in the Western Cape Province, between 1984 and 1992. Patient samples [plasma, serum and peripheral blood mononuclear cells (PBMCs)] were stored at −20 °C. In this study, we selected to sequence the NFLG of five previously identified HIV-1 subtype B samples from the start of the epidemic in South Africa.

### PBMC cultures

PBMC cultures were performed as previously described^26^. Briefly, lymphocytes were separated from the buffy layer on a ficoll gradient and cultured at 37 °C in an atmosphere of 5% CO_2_. The RPMI-1640 medium was supplemented with 20% fetal calf serum, 0.2% Na(HCO_3_)_2_, 2% L-glutamine, antibiotics, and 10–20% Interleukin 2. These PBMCs were co-cultured using umbilical cord lymphocytes that were stimulated with Phytohaemagglutinin for 3 days and treated with Polybrene for 30 minutes before co-cultivation. The medium was replaced every 2 to 3 days and the cell cultures were examined daily for cytopathic effect (CPE).

### Reverse transcriptase assay

The reverse transcriptase (RT) assays were performed as previously described^27^. Briefly, RT activities were assayed in cell-free supernatants of control and infected PBMC cultures. Aliquots of 5 to 20 ml of supernatant fluid were clarified by centrifugation at 10 000 g for 10 minutes, thereafter pelleted at 100 000 g for 50 minutes. The pellets were re-suspended in 60 µl of lysis buffer (10 Mm Tris, 0.2% Triton X-100, 1 mM EDTA, 5 mM dithiothreitol, and 60 mM KCI, pH 7.3). The assays were performed in duplicate on 30 µl amounts of pellet lysate. The template used was 0.01 mg/ml poly(rA) oligo(dT) (Pharmacia, Sweden) in 5 mM MgCl_2_, 2.4 mM Dithiothreitol, 60 mM KCL and 0.0034 mM methyl [H]thymidine-5′ –triphosphate (48 CI/mMole) (Amersham, England).

### Nucleic acid extraction

High molecular weight DNA was extracted from the HIV-1 positive cultures and PBMCs using conventional phenol-chloroform extraction methods and stored at 4 °C.

### PCR amplification

**Figure 3:**
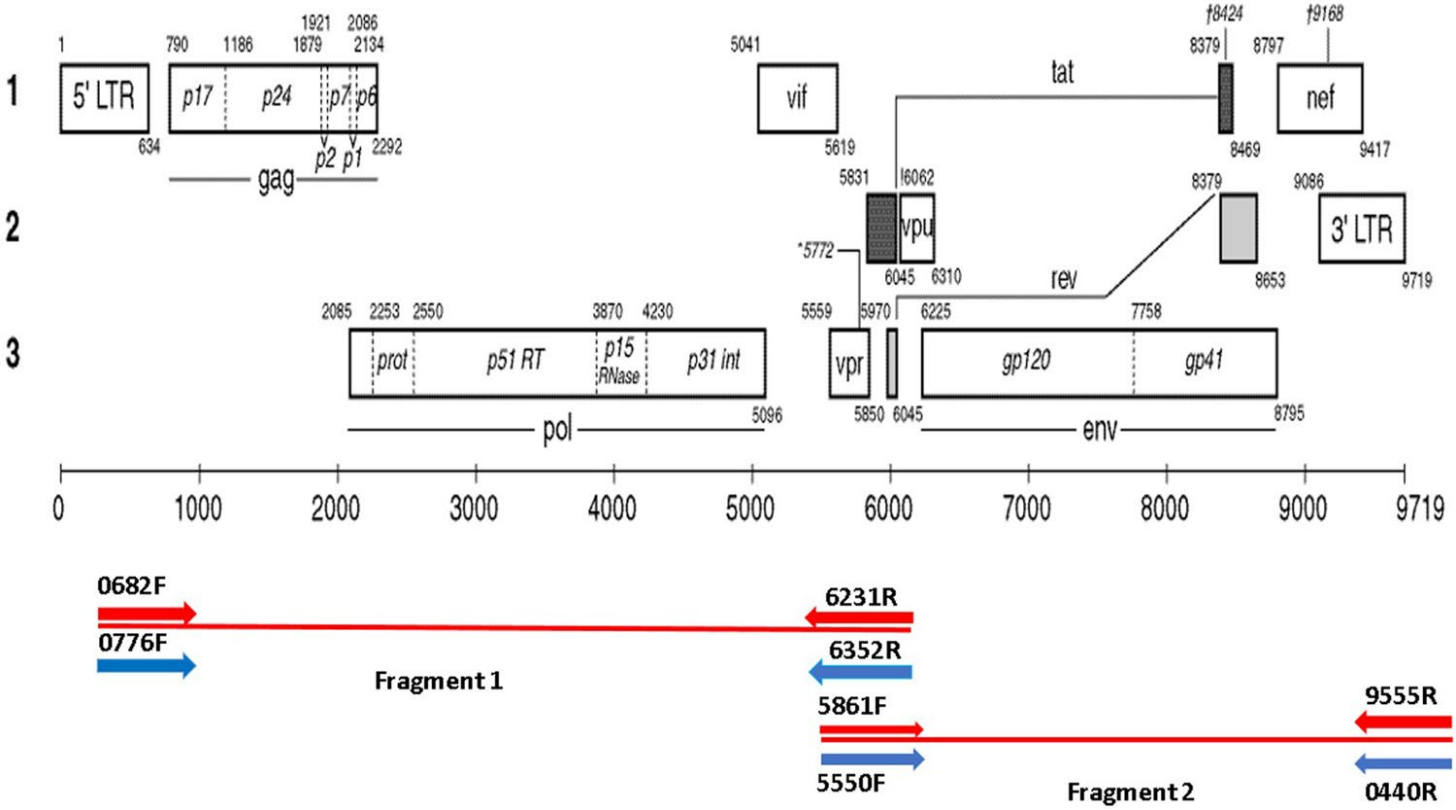
The amplification strategy. The diagram shows amplification for whole genome. Full genome amplification was achieved by amplifying DNA in two fragments. Four primers were used for each fragment utilising pre-nested and nested PCR. Fragment 1 consisted of genes starting from *gag* to *vpu* and was approximately 5.5 Kb in length. The second fragment of genes starting from *vpr* until the 3′LTR and was approximately 3.7 kb in length.

The NFLG amplification protocol was adapted from Grossman *et al*.^28^. Briefly, two overlapping fragments were amplified using the high fidelity KAPA HiFi HotStart ReadyMix (2X) (KAPA Biosystems, USA). The first fragment (F1) ranges from *gag* to *vpu*, position 0776–6231, approximately 5.5 kilobases [kb] in length, relative to HXB2 (Fig. 3). First round PCR was performed with the primer pair 0682F (5′-TCTCTCGACGCAGGACTCGGCTTGCTG-3′) and 6352R (5′-GGTACCCCATAATAGACTGTRACCCACAA-3′), followed by second round nested primer pair with 0776F (5′-CTAGAAGGAGAGAGAGATGGGTGCGAG-3′) and 6231R (5′-CTCTCATTGCCACTGTCTTCTGCTC-3′)^28^. The PCR cycling conditions were as follows: Initial denaturation of 95 °C for 5 minutes, followed by 30 cycles of 98 °C for 20 seconds, 65 °C for 15 seconds and 72 °C for 3 minutes and a final extension at 72 °C for 5 minutes. The second fragment (F2) starts from the *vif* to the 3′LTR, 5861 to 9555, approximately 3.7 kb in length, relative to HXB2. The F2 PCR was performed with first round primers 5550F (5′-AGARGAYAGATGGAACAAGCCCCAG-3′) and 9555R (5′-TCTACCTAGAGAGACCCAGTACA-3′), followed by second round nested PCR with primers 5831F (5′-TGGAAGCATCCRGGAAGTCAGCCT-3′) and 0440R (5′-CCAGAGCTCACCTAGCACCATCCAAAGGTCAGTGGG-3′). The PCR cycling conditions were as follows: Initial denaturation of 95 °C for 5 minutes, followed by 30 cycles of 98 °C for 20 seconds, 65 °C for 15 seconds and 72 °C for 2 minutes and final extension at 72 °C for 5 minutes. Each PCR reaction contained 25 µl of KAPA HiFi HotStart ReadyMix (2X), 1.5 µl of each primer (10 μm), 5 µl of template DNA at a concentration of 20 ng/µl and 17 µl of nuclease-free water to a final volume of 50 µl.

### PCR purification

PCR products were purified using the direct QIAquick PCR purification kit. The QIAquick gel extraction kit was used where multiple bands were visible on the agarose gel (Qiagen, Germany). Purification steps were performed according to the manufacturer’s instructions.

### Sequencing

Conventional Sanger DNA sequencing reactions were performed with the ABI Prism Big Dye Terminator Cycle sequencing kit version 3.1 and run on the ABI 3130xl automated DNA sequencer (Applied Biosystems, USA). The sequencing reactions were performed according to the manufacturer’s instructions. Sequence chromatograms were assembled into contiguous fragments and confirmed in Sequencher version 5.0, https://www.genecodes.com/ (Gene Codes Corporation, Ann Arbor, MI, USA). Sequencher® version 5.4.6 DNA sequence analysis software, Gene Codes Corporation, Ann Arbor, MI USA http://www.genecodes.com.

### Sequence quality control

The nucleotide sequences were verified for stop codons, insertion and deletions using an online quality control program on the HIV LANL database (https://www.hiv.lanl.gov/content/sequence/QC/index.htm). Each patient’s sequences were named according to the 2-letter country code.year.name, for example ZA.85.R1296. HIV BLAST was used to find sequences similar to our sequences https://www.hiv.lanl.gov/content/sequence/BASIC_BLAST/basic_blast.html. Thereafter, patient’s HIV sequences were preliminary characterized and subtyped using the jumping profile Hidden Markov Model (jpHMM) (http://jphmm.gobics.de/)^29^ and Recombinant Identification Program (RIP) version 3.0 https://www.hiv.lanl.gov/content/sequence/RIP/RIP.html^30^.

### Maximum likelihood (ML) phylogenetic tree inference

The HIV-1 subtype reference sequence dataset from 2010 were acquired from e LANL https://www.hiv.lanl.gov/content/sequence/NEWALIGN/align.html. Additional subtype B sequences from 1978–1985 were included in our analyses, as the BLAST results indicated close similarity to our new subtype B sequences. All near-complete genome sequences for subtype B and D from South Africa were also included.

Multiple sequence alignments were done with MAFFT version 7.388^31,32^, as implemented in Geneious version 11.1.5 (https://www.geneious.com). This multiple alignment was further refined by using the HMM option in HIVAlign (https://www.hiv.lanl.gov/content/sequence/VIRALIGN/viralign.html) and manually checked. The best fitting evolutionary model of nucleotide substitution was estimated in Mega version 6.06^33^, using 24 different nucleotide substitution models. The model with the lowest Bayesian Information Criterion (BIC) score was used for further analysis. Subsequently the Maximum Likelihood tree was inferred with the general time reversible (GTR) model of nucleotide substitution^34^, using a discrete Gamma distribution (+G) with five rate categories and by assuming that a certain fraction of sites are evolutionarily invariable (+I)^35^. To test the reliability of the inferred ML tree, we used bootstrap analysis with a total of 100 replicates.

### Recombination analyses

Based on the breakpoints identified with the jpHMM online tool, ML tree topologies were inferred for each of the recombinant fragments of R605. Each fragment was aligned with the HIV-1 subtype reference alignment in MAFFT 7.388^33,34^, as implemented in Geneious 11.1.5 (https://www.geneious.com). ML phylogenetyic trees were inferred with MEGA 6.06 as described before.

### GenBank

The sequences analysed from the study have been submitted to Genbank and are available under the following accession number: MH234639, MH234640, MH234641, MH234642 and MH234643.
